# Genetic parameters for feed efficiency, growth performance, and feeding behaviors traits in Canadian Duroc pigs

**DOI:** 10.1093/jas/skag023

**Published:** 2026-02-02

**Authors:** Duy Ngoc Do, Mohsen Jafarikia, Laurence Maignel, Dan Tulpan, Deborah Adewole, Justin Holl, Brian Sullivan, Younes Miar

**Affiliations:** Department of Animal Science and Aquaculture, Dalhousie University, Bible Hill, NS B2N 5E3, Canada; Department of Animal Science and Aquaculture, Dalhousie University, Bible Hill, NS B2N 5E3, Canada; Canadian Centre for Swine Improvement, Ottawa, ON K1V 0M7, Canada; Department of Animal Biosciences, University of Guelph, Guelph, ON N1G 2W1, Canada; Canadian Centre for Swine Improvement, Ottawa, ON K1V 0M7, Canada; Department of Animal Biosciences, University of Guelph, Guelph, ON N1G 2W1, Canada; Department of Animal and Poultry Science, University of Saskatchewan, Saskatoon, SK S7N 5A8, Canada; The Pig Improvement Company, Genus plc, Hendersonville, TN 37075, United States; Canadian Centre for Swine Improvement, Ottawa, ON K1V 0M7, Canada; Department of Animal Science and Aquaculture, Dalhousie University, Bible Hill, NS B2N 5E3, Canada

**Keywords:** feed efficiency, genetic correlations, heritability, pigs, feed conversion ratio, residual feed intake

## Abstract

Feed represents the largest cost in pig production, making improvement in feed efficiency (FE) essential for maximizing the profitability for pig producers. The objective was to model different FE traits and to estimate the phenotypic and genetic correlations among these FE traits, growth traits, and feeding behavior traits. The FE traits included feed conversion ratio (FCR), six measures of residual feed intake (RFI1-6), residual gain (RG), residual intake and gain (RIG), and Kleiber ratio (KR). The RFIs were calculated by regressing daily feed intake (DFI) on different combinations of covariates, including metabolic midweight, average daily gain (ADG120), backfat thickness (BF120), and loin depth (LD120). Feeding behavior traits analyzed were the number of visits to the feeder per day (NVD), total time spent eating per day (TPD), feed intake rate (FR), feed intake per visit (FPV), and time spent eating per visit (TPV). Genetic parameters of the studied traits from 14,939 Duroc pigs were estimated using ASReml-R version 4.2. The univariate model indicated that fixed effects (sex, herd-year-season), covariates (initial body weight, adjusted age at 120 kg), and random effects (pen, litter, maternal genetics) are all significant for FE and performance traits (*P* < 0.05). Heritability estimates (±SE) for FE traits ranged from 0.28 ± 0.03 for RFI1 to 0.34 ± 0.03 for FCR indicating moderate heritability. Similarly, most of the performance and feeding behavior traits had moderate heritability, with higher estimates observed for BF120 (0.51 ± 0.03), FR (0.62 ± 0.02) and TPD (0.60 ± 0.02). All six RFI traits had weak to moderate positive genetic correlations (±SE) with BF120 (from 0.13 ± 0.06 for RFI6 to 0.58 ± 0.04 for RFI1 and RFI2). Only RFI1, RFI2, and RFI3 had significant genetic correlations with LD120 estimated at -0.40 ± 0.05, -0.41 ± 0.05 and -0.23 ± 0.06, respectively. FCR displayed significant genetic correlations with ADG120 (-0.64 ± 0.07), BF120 (0.55 ± 0.05), and LD120 (-0.61 ± 0.07). RG and RIG had significant positive genetic correlations with ADG120 and low or non-significant genetic correlations with BF120 and LD120. FE traits had weak genetic correlations with feeding behavior traits. Overall, the results demonstrate that RFI is a more reliable and advantageous trait for improving FE compared with FCR, as it maintains favorable relationships with growth traits while minimizing negative effects on carcass quality. The weak associations between FE and feeding behavior traits further suggest that selecting for RFI can enhance feed utilization without unintended behavioral consequences. These findings highlight the potential of RFI as a selection criterion to advance genetic improvement programs focused on sustainable production efficiency without compromising key economic and quality traits.

## Teaser

Feed is the biggest expense in pig farming, making feed efficiency is among the top priorities for producers. This study shows that selecting pigs based on residual feed intake (RFI) can improve efficiency without compromising growth or meat quality.

## Introduction

Improving feed efficiency without compromising other production traits is one of the major targets of pig producers since feed is the most significant cost in pig production (Patience et al. 2015). In fact, improving feed efficiency increases industry competitiveness, decreases demand for global feed resources, and complements environmental sustainability (Soleimani and Gilbert 2020; Dekkers 2025). For instance, pigs selected for low residual feed intake (RFI) (more efficient, LRFI) vs. high residual feed intake (less efficient, HRFI) found that LRFI pigs produced 7% lower climate change impact (2.60 vs. 2.77 kg CO_2_-eq/kg live pig at farm gate), 8% lower eutrophication potential (3.35 vs. 3.63 g P-eq), 6% lower land occupation, and had similar reductions in acidification and water depletion, which indicated that genetic selection for feed efficiency, translates into measurable environmental improvements (Soleimani and Gilbert 2020). Different approaches have been used to improve feed efficiency and selective breeding is one of the sustainable solutions since it passes the genetic merits to the next generations (Davoudi et al. 2022). Improving feed efficiency through genetic selection requires reliable and easy-to-implement indicators. Currently, feed conversion ratio (FCR) and RFI are commonly used indicators of feed efficiency in pigs (Díaz et al. 2017; Gilbert et al. 2017; Davoudi et al. 2022). FCR is computed as the ratio of total feed intake to total weight gain. FCR is used to improve feed efficiency in several breeding companies since it is directly calculated from feed intake and performance data. FCR is straightforward to calculate and easy to explain to producers (Patience et al. 2015). Nevertheless, FCR is a ratio trait, and selecting for improved FCR can inadvertently impact other economically important traits, such as backfat (BF) or average daily gain (ADG). Koch et al. (1963) proposed RFI as an alternative measure, which is defined as the difference between the observed feed intake and the expected feed intake for a given production. RFI has been estimated using linear models of varying complexity after that, from simple regressions with Daily feed intake (DFI) and ADG to more comprehensive models including fixed effects and covariates, such as sex, body weight, BF, metabolic mid-weight (MMW), and loin depth (LD) (Hoque and Suzuki 2008; Do et al. 2013; Lu et al. 2017; Sanntiago et al. 2021b). Given that previous studies reported substantial differences in model performance, multiple model structures to identify the best approach for accurate RFI prediction.

Besides RFI, Berry and Crowley (2013) proposed two alternative indicators of feed efficiency. The first is residual body weight gain (RG), defined as the residual from a multiple regression model of ADG on both dry matter intake (DMI) and BW. The second is residual intake and gain (RIG), which combines favorable characteristics of both RFI and RG. Several indirect measures of feed efficiency have also been proposed, such as relative growth rate, which reflects growth relative to instantaneous body size (Fitzhugh and Taylor 1971), and the Kleiber ratio, defined as ADG per unit of metabolic BW (Kleiber 1961). Both measures can be considered feed efficiency indicators provided that all animals on the test are fed the same restricted diet (Berry and Crowley 2013). Therefore, these measures are rarely used in breeding programs, and most livestock breeding programs use either FCR or RFI as an indicator of feed efficiency. Many studies have been devoted to modelling RFI and predicting breeding values for RFI and other feed efficiency indicators in pigs. Heritability estimates for these traits vary between studies and breeds, with RFI heritability ranging from 0.1 in Large White pigs (Johnson et al. 1999) to 0.4 in Duroc pigs (Hoque and Suzuki 2008).

Previous studies indicated that feeding behavior plays an important role in pig production and feed efficiency, directly influencing growth performance and nutrient utilization (Carcò et al. 2018; Piles et al. 2025). Variations in feeding rate, meal size, frequency, and duration affect feed intake and body composition, which in turn impact growth rates and carcass quality. Studies show that pigs with faster feeding rates tend to have higher growth rates, greater protein and fat retention, and improved carcass traits (Carcò et al. 2014; Piles et al. 2025). In addition, feed efficiency traits are also correlated with feeding behaviors. For example, DFI shows positive correlations with FCR (genetic: 0.65; phenotypic: 0.67) and RFI (genetic: 0.95; phenotypic: 0.90) (Do et al. 2013). Identification of correlations between feeding behavior and feed efficiency is important for dissecting the molecular mechanisms and genetic basis underlying feed efficiency and setting up a selection program for feed efficiency. There are no studies on model RFI, RG, and RIG in Canadian Duroc pigs, and the majority of other studies for RFI in other countries are based on Duroc boars. In addition, only a few studies have provided a comprehensive measure of feeding behaviors and their correlations with growth performance and feed efficiency traits. It is important to examine the potential of different feed efficiency indicators, their correlation with feeding behaviors and performance traits. The objective of the current study is to model RFI, RG, and RIG, compare the results with FCR, and estimate their correlations with other production traits and feeding behaviors in both male and female Canadian Duroc pigs.

## Materials and methods

Data for this study was provided by the Pig Improvement Company (PIC, Manitoba, Canada), and pigs were cared for according to the Canadian Council of Animal Care guidelines (CCAC 1993). Since data were collected as part of routine commercial operations, approval by an Animal Care and Use Committee was not required.

### Animals and phenotypes

A total of 15,423 pigs having both feeding records and performance measures were used in the current study. Daily feed intake was measured using automatic feeding systems (FIRE^®^ feeders, Osborne Industries Inc., or IVOG^®^ feeders, Hokofarm Group) with one feeder installed per pen. Feed intake data were collected from 324 pens across 13 PIC farms in Quebec between 2015 and 2022. Data covered all four seasons: spring (March–May), summer (June–August), autumn (September–November), and winter (December–February). Each herd contained 20–37 pens. During testing, these pigs were fed *ad libitum* using a commercial finisher diet formulated to meet the nutritional requirements for optimal growth and carcass quality. The diet typically consisted of corn (60–70%), soybean meal (15–25%), wheat middlings or distillers dried grains with solubles (5–10%), and a vitamin-mineral premix (1–2%) (NRC 2012; PIC 2021). The average weight and age of the pigs at the start of the testing was 35.6 ± 0.02 kg and 74.71 ± 0.06 days, respectively. When the pigs reached approximately 120 kg, they were sent to a processing plant unless selected for breeding. The daily records were processed using the standardized procedures described by Casey et al. (2005). Each electronic feeder visit was screened for errors, such as implausible intake values, unrealistic durations, duplicate entries, and missing data. Erroneous records were corrected or removed, and pigs with frequent errors were replaced with error-free records to maintain dataset integrity. This pre-cleaning ensured that estimates of feed intake and feeding behavior were biologically meaningful and suitable for subsequent modeling. For calculation of feed efficiency and feeding behaviors, only animals with a minimum of 60 consecutive days of feed intake records were kept. Additionally, only animals whose start and final weights were recorded within two days of their respective feed period start and end dates and that were housed in pens with at least eight animals were included. After filtering, a total of 14,939 pigs (5,426 females and 9,513 males) with at least one phenotype were retained for feed efficiency modelling and feeding behavior calculation.

For calculation of feed efficiency and feeding behaviors, only animals with a minimum of 60 consecutive days of feed intake records were kept. Additionally, only animals whose start and final weight were recorded within two days of their respective feeding period start and end dates and that were housed in pens with at least eight animals were included. The formulas from the Canadian Swine Improvement Program were used for adjusting the ages and measuring traits to 120 kg of body weight as described below:

Ages adjusted to 120 kg of body weight (A120, days) were calculated using the following formula, which is a function of age (A; days) and weight (W; kg) at scanning:


$${\text{A}}_{120}=A-\frac{W-120}{b\;\times \;\frac{W}{A}},$$

where b is 1.826 for males and 1.715 for females (Tang et al. 2019), respectively.

BF thickness (BF120) adjusted to 120 kg was computed based on the measured BF thickness (mm) and W (kg) using the following formula:


$$\mathrm{BF}120=\mathrm{Actual}\;\mathrm{BF}\times \frac{A_{\mathrm{BF}120}}{A_{\mathrm{BF}120}+B_{\mathrm{BF}120}\times (W-120)},$$

where $A_{\mathrm{BF}120}$ and $B_{\mathrm{BF}120}$ are adjustment factors with values of 12.44 and 0.087 for males, and 13.35 and 0.095 for females, respectively.

Lean depth adjusted to 120 kg (LD120) was computed as a function of measured lean depth (mm) and body weight at scanning (W in kg), using the following formula:


$$\mathrm{LD}120=\mathrm{Actual}\;\mathrm{LD}\times \frac{A_{\mathrm{LD}120}}{A_{\mathrm{LD}120}+B_{\mathrm{LD}120}\times (W-120)},$$

where $A_{\mathrm{LD}120}$ and $B_{\mathrm{LD}120}$ are 67.9 and 0.2817 for males, and 69.34 and 0.2514 for females, respectively.

Average daily gain (ADG120, kg) was calculated from the beginning to the end of the testing periods as follows:


$$\mathrm{ADG}120=\frac{120-\mathrm{IBW}}{A120},$$

where IBW is the initial body weight at the beginning of the testing period.

Daily feed intake (DFI; kg) was calculated using the following formula:


$$\mathrm{DFI}=\frac{\mathrm{Total}\;\mathrm{amount}\;\mathrm{of}\;\mathrm{feed}\;\mathrm{intake}\;\mathrm{during}\;\mathrm{testing}\;\mathrm{period}}{A120-\mathrm{age}\;\mathrm{the}\;\mathrm{begining}\;\mathrm{of}\;\mathrm{the}\;\mathrm{test}},$$

Using the ADG120 and DFI, FCR (kg/kg) was calculated using the following formula:


$$\mathrm{FCR}=\frac{\mathrm{DFI}}{\mathrm{ADG}120},$$

The mid-test metabolic BW (MMW) was required to calculate the Kleiber ratio and RFI measures. It was calculated as follows:


$$\mathrm{MMW}={\left(\frac{\mathrm{IBW}+\mathrm{body}\;\mathrm{weight}\;\mathrm{at}\;\mathrm{the}\;\mathrm{end}\;\mathrm{of}\;\mathrm{testing}\;\mathrm{period}}{2}\right)}^{0.75},$$

where IBW is the initial body weight at the beginning of the testing period.

Using the ADG120 and MMW, the Kleiber ratio (Kleiber 1961) was calculated using the following formula:


$$\mathrm{KR}=\frac{\mathrm{ADG}120}{\mathrm{MMW}}.$$

RFI was estimated using the following linear regression models, which were based on the previous studies (Koch et al. 1963, Hoque et al. 2008; Do et al. 2013; Gilbert et al. 2017; Lu et al. 2017; Santiago et al. 2021b):

Model1 (RFI1):


$$\mathrm{RFI}1=\;\mathrm{DFI}-(\beta_{0}+\beta_{1}I\mathrm{BW}+\beta_{2}A\mathrm{DG}120),$$

Model 2 (RFI2):


$$\mathrm{RFI}2=\mathrm{DFI}-(\beta_{0}+\beta_{1}I\mathrm{BW}+\beta_{2}A\mathrm{DG}120+\beta_{3}M\mathrm{MW}),$$

Model 3 (RFI3):


$$\mathrm{RFI}3=\mathrm{DFI}-(\beta_{0}+\beta_{1}I\mathrm{BW}+\beta_{2}A\mathrm{DG}120+\beta_{4}BF120),$$

Model 4 (RFI4):


$$\mathrm{RFI}4=\;\mathrm{DFI}-{(\beta }_{0}+\beta_{1}I\mathrm{BW}+\beta_{2}A\mathrm{DG}120+\beta_{3}M\mathrm{MW}+\beta_{4}BF120),$$

Model 5 (RFI5):


$$\mathrm{RFI}5={\mathrm{DFI}-(\beta }_{0}+\beta_{1}I\mathrm{BW}+\beta_{2}A\mathrm{DG}120+\beta_{3}M\mathrm{MW}+\beta_{5}LD120),$$

Model 6 (RFI6):


$$\mathrm{RFI}6={\mathrm{DFI}-(\beta }_{0}+\beta_{1}I\mathrm{BW}+\beta_{2}A\mathrm{DG}120+\beta_{3}M\mathrm{MW}+\beta_{4}BF120+\beta_{5}LD120),$$

where $\beta_{0}$ is defined as the intercept, β1, $\beta_{2}$, $\beta_{3}$, $\beta_{4,}$ and $\beta_{5}$ are the partial regression coefficients of IBW, ADG120, MMW, BF120, and LD120, respectively.

RG was estimated using the following linear regression model:


$$\mathrm{RG}={\mathrm{ADG}120-(\beta }_{0}+\beta_{1}D\mathrm{FI}+\beta_{2}M\mathrm{MW}+\beta_{3}BF120+\beta_{4}LD120),$$

where $\beta_{0}$ is defined as the intercept $\beta_{1}$, $\beta_{2}$,${\;\beta }_{3,}\;$and $\beta_{4}\;$are the partial regression coefficients of ADG120 on DFI, MMW, BF120, and LD120, respectively. Finally, ϵ represents RG.

Residual intake and gain (RIG) was calculated based on the method proposed by Berry and Crowley (2013) as follows:


$$\mathrm{RIG}=(-1\;\times (\frac{{\mathrm{RFI}6}_{i}}{{\mathrm{RFI}6}_{\mathrm{sd}}}))+\;(\frac{{\mathrm{RG}}_{i}}{{\mathrm{RG}}_{\mathrm{sd}}}),$$

where RFI6_i_ is the RFI6 for animal *i*, RFI6_sd_ is the standard deviation of RFI6 for all animals, RG_i_ is the residual gain of animal *i*, and RG_sd_ is the standard deviation of RG for all animals.

For each pig, the feeding behavior traits included total time spent eating per day (TPD; min/day), number of visits to the feeder per day (NVD, visits/day), time spent eating per visit (TPV; min/visit), which was calculated as TPD/NVD, mean feed intake per visit (FPV; kg/visit), and mean feed intake rate (FR; g/min), which was calculated as DFI/TPD. After all traits were computed, potential outliers were identified and removed by considering phenotypic records, which were more than five standard deviations from the mean as missing values. The number of excluded values for each trait ranged from 0 (for DFI and KR) to 61 (NVD) (Table 1).

**Table 1:** Descriptive statistics for feed efficiency, growth, and feeding behavior traits in Duroc pigs.

Traits	Abbs	Unit	Count	Mean	Min	Max	SD	SE	CV(%)
**Average daily gain**	ADG120	kg	14,934	1.09	0.68	1.55	0.09	0.001	8.50
**Backfat thickness**	BF120	mm	14,925	11.49	4.54	23.55	2.51	0.021	21.80
**Loin depth**	LD120	mm	14,937	73.40	48.87	94.12	5.95	0.049	8.10
**Daily feed intake**	DFI	kg	14,939	2.47	1.09	3.97	0.32	0.003	12.80
**Feed conversion ratio**	FCR	kg/kg	14,936	2.26	1.30	3.35	0.23	0.002	10.00
**Residual feed intake1**	RFI1	kg	14,935	0.01	-1.00	1.10	0.22	0.002	
**Residual feed intake2**	RFI2	kg	14,936	0.00	-1.05	1.04	0.21	0.002	
**Residual feed intake3**	RFI3	kg	14,931	0.01	-0.96	1.01	0.21	0.002	
**Residual feed intake4**	RFI4	kg	14,929	0.00	-0.91	0.90	0.19	0.002	
**Residual feed intake5**	RFI5	kg	14,931	0.01	-0.96	0.99	0.21	0.002	
**Residual feed intake6**	RFI6	kg	14,929	0.00	-0.90	0.92	0.19	0.002	
**Residual gain**	RG	kg	14,931	0.00	-0.31	0.34	0.07	0.001	
**Residual intake and gain**	RIG	kg	14,930	0.01	-7.82	7.19	1.57	0.013	
**Kleiber ratio**	KR		14,939	0.04	0.03	0.06	0.00	0	7.20
**Time spent in feeder per day**	TPD	min	14,934	64.01	15.77	125.78	12.46	0.102	19.50
**Number of visits to feeder per day**	NVD	count	14,878	11.63	2.19	41.77	5.54	0.045	47.60
**Time spent per visit**	TPV	min	14,907	6.57	0.65	22.94	3.16	0.026	48.10
**Feeding rate**	FR	kg	14,934	0.04	0.01	0.08	0.01	0	22.50
**Feed eaten per visit**	FPV	kg/min	14,911	0.26	0.04	0.88	0.12	0.001	47.90

Abbs, abbreviations; Count, units, number of animals per trait; Mean, means; Min, minimum; Max, maximum; SD, standard deviations; SE, standard error; and CV, coefficient variation values.

### Statistical and genetic analysis

Akaike information criterion (AIC) and adjusted R^2^ were used to select the best-fitted model for RFI. The model with the lowest AIC score or highest adjusted R^2^ values was identified as the best model for RFI. To determine the optimal animal model for variance component estimation, the significance of fixed and random effects was tested using univariate models. The year and season of birth were combined with the testing herd (when pigs were tested) to form a fixed effect of herd-year-season. The model included herd-year-season and sex as fixed effects, with initial body weight and adjusted age at 120 kg body weight as covariates. The significance (*P* < 0.05) of fixed effects was statistically tested using the Wald test in ASReml-R version 4.2 (Butler et al. 2017). For each trait, the significance of each random effect was tested by comparing the full and reduced models as follows:


$$\begin{array}{c} -2({\mathrm{logL}}_{\mathrm{reduced}\;\mathrm{model}}-{\mathrm{logL}}_{\mathrm{full}\;\mathrm{model}})\\ ∼x_{df\;(\mathrm{full}\;\mathrm{model})-df(\mathrm{reduced}\;\mathrm{model})}^{2}, \end{array}$$

where *logL* represents log-likelihood and *df* is the number of degrees of freedom.

The variance components were estimated for each trait using the following univariate model:


y=Xb+Za+Sc+Wp+Mm+e,

in which ***y*** is the vector of phenotypic observations, ***b*** is the vector of fixed effects, ***a*** is the vector of random additive genetic effects, ***c*** is the vector of random common litter effects, ***p*** is the vector of random pen effect, ***m*** is the vector of random maternal genetic effects, and ***e*** is a vector of residual effects. The ***X***, ***Z***, ***S***, ***W*,** and ***M*** are the incidence matrices associating the phenotypic observations to the fixed effects, random additive genetic effects, random common litter effects, random pen effects, and random maternal genetic effects, respectively. It was assumed that random effects were independent and normally distributed.


[acpme]∼N[Aσa200000Iσc200000Iσp20000 0Aσm2 00000Iσe2],

where $\sigma_{a}^{2}$, $\sigma_{c}^{2}$, $\sigma_{p}^{2}$, $\sigma_{m}^{2}$, and $\sigma_{e}^{2}$ are the variances of random additive genetic, common litter, pen, maternal, and residual effects, respectively, ***I*** is an identity matrix and ***A*** is the numerator relationship matrix. The pedigree of phenotyped animals contained 24 generations, comprising 116,221 individuals (1,134 sires and 5,634 dams) to construct the numerator relationship matrix. All analyses used ASReml-R version 4.2 (Butler et al. 2017).

The model applied for bivariate analyses between each pair of traits was:


[y1y2]= [ X100X2 ] [b1b2]+ [ Za100Za2 ] [a1a2]+[ Sc100Sc2 ] [c1c2]+ [ Wp100Wp2 ][p1p2]+[Mm100Mm2 ] [m1m2]+ [e1e2],

where $y_{1}$ and $y_{2}\;$are the vectors of observations for the first and second traits; $b_{1}$, $b_{2}$, $a_{1}$, $a_{2}$, $c_{1}$, $c_{2},\;{\;p}_{1}$, $p_{2}$, $m_{1},\;m_{2},\;e_{1}$, and $e_{2}$ are the vectors of fixed, random additive genetic, common litter, pen, and residual effects for traits 1 and 2, respectively; and $X_{1}$, $X_{2}$, ${\;Z}_{a1}$, $Z_{a2}$, ${\;S}_{c1}$, $S_{c2}$, $Z_{p1}$, $Z_{p2}$, $M_{m1}$, and $M_{m2}$ are the matrices associating observations to fixed, random additive genetic, common litter, pen, and maternal genetics effects for traits 1 and 2, respectively. The random effects were assumed to be normally distributed with a mean of zero and a (co)variance structure equal to:


[a1a2] ∼ (0, A⊗ [σa12σa1a2σa1a2σa22] ),

[c1c2] ∼ (0, I⊗ [σc12σc1c2σc1c2σc22] ),[p1p2] ∼ (0, I⊗ [σp12σp1p2σp1p2σp22] ),[m1m2] ∼ (0, A⊗ [σm12σm1m2σm1m2σm22] ), and[e1e2] ∼ (0, I⊗ [σe12σe1e2σe1e2σe22] ),

where ***A*** is the numerator relationship matrix; ***I*** is an identity matrix; $\sigma_{a1}^{2}$, $\sigma_{a2}^{2}$, $\sigma_{c1}^{2}$, $\sigma_{c2}^{2},\;\sigma_{p1}^{2}$, $\sigma_{p2}^{2}$, $\sigma_{m1}^{2}$, $\sigma_{m2}^{2},\;\sigma_{e1}^{2}$, and $\sigma_{e2}^{2}$ are the variances of random additive genetic, common litter, pen, maternal genetics, and residual effects for traits 1 and 2, respectively; $\sigma_{a1a2}$, $\sigma_{c1c2}$, $\sigma_{p1p2}$, $\sigma_{m1m2}$, and $\sigma_{e1e2}$ are the covariances of additive genetic, common litter, pen, maternal genetics, and residual effects between traits 1 and 2, respectively.

Phenotypic and genetic correlations among traits were estimated pairwise in bivariate models. The significance of these estimates was tested using a Z-test with the null hypothesis that the estimates are equal to zero for a confidence level α of 0.05.

## Results and discussion

### Descriptive statistics and models of RFI

The descriptive statistics for the studied traits are shown in Table 1. The studied pigs had an average ADG120 of 1.09 kg, an average DFI of 2.47 kg, and an average FCR of 2.26. Feed efficiency indicators calculated from linear regression models (RFIs, RG, and RIG) had mean values of zero (or close to zero), as expected. On average, pigs spent approximately one hour (64.01 min) per day at the feeder, and they had 11.63 daily visits. The coefficient of variation (CV) ranged from 7.2% (KR and ADG120) to 48.1% (NVD).

The studied population had higher ADG120 and lower BF120 compared to Korean Duroc pigs (1.07 kg and 14.23 m, Santiago et al. 2021b), Chinese Duroc pigs (0.84 kg/day and 12.23 mm, Li et al. 2022), and Duroc pigs raised in the USA (0.87 [kg] and 11.03 mm, Lu et al. 2017). DFI reported in the current study (2.47 kg) was similar to the values of 2.48 kg (Santiago et al. 2021a) or 2.40 kg (Do et al. 2013) in Duroc breeds raised in Korea and Denmark, respectively. The studied population also demonstrated better FCR compared to Korean Duroc pigs, which had an FCR of 2.30 kg feed/kg gain (Santiago et al. 2021b), and Chinese pigs with an FCR of 2.70 kg feed/kg gain (Li et al. 2022). The lower BF120 in the studied population could reflect selection for leaner genotypes, which is often associated with higher ADG120 due to efficient partitioning of energy toward muscle rather than fat. The similarity in DFI across populations suggests that feed intake is less sensitive to genetic divergence and more influenced by standardized feeding regimes or appetite regulation mechanisms. This improved FCR could result from genetic selection for feed efficiency, optimized nutrition, or management practices that enhance nutrient absorption and growth in the studied population.

By definition, the sum of residuals in a regression model is zero, which means their mean is also zero (or very close to zero in practice due to rounding or numerical precision). Since RFI, RG, and RIG are computed as residuals from models that regress feed intake, body weight gain, or a combination of both on relevant predictors (eg, metabolic body weight, growth rate), their mean phenotypic values should theoretically be zero. This is consistent with the findings in the cited studies (Hoque et al. 2009; Do et al. 2013; Santiago et al. 2021b). Regarding feeding behaviors, Do et al. (2013) reported higher mean TPD values of 81.05 min/day and lower mean NVD values of 8.58 min/visit in boars compared to the values obtained in this study. Conversely, Lu et al. (2017) reported lower mean TPD (61.92 min/day) and NVD (5.77 visits/day) in Duroc pigs raised in the USA compared to our results. This high variability in NVD suggests individual differences in feeding strategies, potentially influenced by genetic, environmental, or social factors within the pen, which could affect energy expenditure and feed efficiency. Differences in the observed mean values across studies may be attributed to various factors, such as the age of animals at the time of measurement, variations in the initial testing weight and testing duration, the methods of adjustment for age of animals and growth, differences in electronic feeders, the number of pigs allowed for each testing pen, and variations in data filtering procedures.

The partial regression coefficients for each covariate used to predict RFIs are shown in Table 2. The adjusted R^2^ ranged from 0.50 (model 1, RFI1) to 0.64 (model 6, RFI6). Among the RFI models, RFI6 had the highest adjusted R^2^ and the lowest AIC indicating, it was the best-fitted model. Since its initial definition by Koch et al. (1963), various linear models have been used to compute RFI. These range from simple models including only DFI and ADG, to more complex models incorporating additional fixed effects and covariates, such as sex, initial body weights and other regressors, such as BF, MMW, and LD. In this study, we evaluated six different combinations of fixed effects and covariates in the models. Hoque et al. (2008) reported comparable adjusted R^2^ values of 0.59, 0.68, 0.61, and 0.69 for models used to calculate RFI1, RFI2, RFI3, and RFI4 in Duroc pigs, respectively. Santiago et al. (2021b) reported higher values of RFI1 to RFI5 from 0.76 (RFI1) to 0.77 (RFI4). Previous studies reported that including BF in the models resulted in the highest R^2^ value, indicating importance of BF in predicting DFI (Hoque et al. 2009; Santiago et al. 2021b). Santiago et al. (2021b) reported that the inclusion of the loin muscle area trait did not significantly affect the RFI model predictions. The partial regression coefficient of ADG in Korean Duroc pigs reported by Santiago et al. (2021b) ranged from 1.52 to 2.04, which is higher than 0.859 (RFI6) to 1.899 (RFI1) found in the current study. The partial regression coefficient for BF (∼ 0.22) was higher than the value reported for Korean Duroc pigs (0.019), but lower than those observed in Korean Landrace (0.37) and Yorkshire (0.33) breeds (Berry and Crowley 2013). In addition to the variables used in this study to predict RFI, de Haer (1992) identified several other factors that may influence RFI prediction, including diet digestibility, variations in energy and nutrient absorption, physical activity allocation, temperature regulation, tissue maintenance, health status, protein and fat efficiency, and basal metabolic rate. We tested various models to predict RFI using the available measurements, evaluating their performance based on AIC and adjusted R^2^. Our analysis identified the model used for RFI6 as the best RFI model in the current population. Similar to our findings, Lu et al. (2017) reported that a model incorporating ADG, BF, MMW, and LD was the most accurate predictor of RFI, as evidenced by the highest adjusted R^2^. In Duroc pigs raised in Korea, Santiago et al. (2021b) reported the model with Initial test age, Initial body weight, ADG, BF, and LD (called RFI4) was the best-fitted model with the highest adjusted R^2^ and the lowest AIC compared to four other models. The selection of the RFI6 model, therefore, represents the optimal balance of predictive power and parsimony for our specific dataset. The coefficients within this best-fitting model are essential for understanding the unique relationships between growth and performance traits and feed intake in this particular Duroc population. These results underscore the need for population-specific modeling approaches to accurately estimate RFI and ensure maximum genetic progress in breeding programs.

**Table 2:** Regression coefficients of covariates, Akaike information criterion and square for different models of residual feed intakes.

RFI definition	Intercept	Weight at beginning of the test	ADG120*	MMW	BF120*	LD120*	AIC	R^2^ (Adjusted)
**RFI1**	-0.090	0.014	1.899				-1,860.709	0.501
**RFI2**	-0.589	0.002	1.076	0.069			-4,010.080	0.566
**RFI3**	-0.360	0.015	1.850		0.026		-3,219.671	0.543
**RFI4**	-1.022	0.000	0.863	0.082	0.033		-6,523.948	0.632
**RFI5**	-0.222	0.015	1.862	0.026		-0.002	-3,269.677	0.545
**RFI6**	-0.782	0.000	0.859	0.033	0.084	-0.004	-6,749.755	0.637

* ADG120: Average daily gain adjusted by 120 kg of body weight; BF120: Backfat thickness adjusted by 120 kg of body weight; LD120: Loin depth adjusted by 120 kg of body weight; MMW: Mid test metabolic weight, AIC: Akaike information criterion; R^2:^ The coefficient of determination, representing the proportion of the total variation in the dependent variable explained by the model.

### Significant effects, variance components, and heritability

Sex, age at the end of the test, and herd-year-season effects significantly impacted all traits, except for NVD, which was not significantly influenced by sex in the univariate models (Table 3). The random pen, maternal genetics, and common litter effects were significantly affecting most of the studied traits (Table 3). Variance components and heritability estimate for the traits are shown in Table 3. The pen effect explained from 0.4% (for TPD) to 23.3% (for RG), and the common litter effect explained from 1.6% (for RFI) to 22.8% (for KR) of the total phenotypic variance of the studies, respectively. Heritability estimates (±SE) for feed efficiency traits were moderate (from 0.281 ± 0.025 for RFI5 to 0.341 ± 0.027 for FCR), while high heritability estimates were reported for BF120 (0.511 ± 0.027), FR (0.621 ± 0.023), and TPD (0.597 ± 0.023).

**Table 3:** Variance components and heritability estimates for feed efficiency, growth performance, and feeding behavior traits in Duroc pigs.

Traits*	σ^2^_p_	σ^2^_c_	σ^2^ _a_	σ^2^_m_	σ^2^_e_	$c_{p}^{2}$	$c_{c}^{2}$	$c_{m}^{2}$	h^2^
**ADG120**	7.00E-05	4.60E-04	4.30E-04	1.20E-04	3.20E-04	0.048 ± 0.008	0.327 ± 0.014	0.087 ± 0.015	0.308 ± 0.026
**BF120**	1.16E-02	1.90E-01	2.47E + 00	9.88E-02	2.06E + 00	0.002 ± 0.001	0.039 ± 0.007	0.020 ± 0.008	0.511 ± 0.027
**LD120**	1.71E-01	1.54E + 00	2.87E + 00	1.93E-01	8.41E + 00	0.013 ± 0.004	0.117 ± 0.010	0.015 ± 0.009	0.218 ± 0.024
**DFI**	1.13E-03	2.26E-03	1.40E-02	1.27E-03	2.05E-02	0.029 ± 0.006	0.058 ± 0.008	0.033 ± 0.010	0.356 ± 0.027
**FCR**	1.26E-03	1.00E-03	1.04E-02	6.40E-04	1.72E-02	0.041 ± 0.008	0.033 ± 0.007	0.021 ± 0.009	0.341 ± 0.027
**RFI1**	1.51E-03	1.17E-03	1.16E-02	7.90E-04	2.02E-02	0.043 ± 0.008	0.033 ± 0.007	0.022 ± 0.009	0.328 ± 0.026
**RFI2**	9.00E-04	8.80E-04	1.14E-02	7.40E-04	1.97E-02	0.027 ± 0.005	0.026 ± 0.007	0.022 ± 0.009	0.339 ± 0.026
**RFI3**	1.55E-03	7.60E-04	8.48E-03	5.90E-04	1.85E-02	0.052 ± 0.009	0.025 ± 0.007	0.020 ± 0.008	0.284 ± 0.025
**RFI4**	8.70E-04	4.90E-04	7.90E-03	5.40E-04	1.79E-02	0.031 ± 0.006	0.018 ± 0.007	0.019 ± 0.008	0.285 ± 0.024
**RFI5**	1.61E-03	7.50E-04	8.39E-03	5.90E-04	1.85E-02	0.054 ± 0.010	0.025 ± 0.007	0.020 ± 0.008	0.281 ± 0.025
**RFI6**	8.80E-04	4.40E-04	7.80E-03	5.50E-04	1.81E-02	0.032 ± 0.006	0.016 ± 0.007	0.020 ± 0.008	0.281 ± 0.024
**RG**	1.10E-04	2.20E-04	3.90E-04	5.00E-05	5.50E-04	0.084 ± 0.014	0.170 ± 0.011	0.037 ± 0.011	0.294 ± 0.027
**RIG**	7.62E-02	5.06E-02	4.12E-01	3.09E-02	8.74E-01	0.053 ± 0.009	0.035 ± 0.008	0.021 ± 0.008	0.285 ± 0.025
**KR**	2.20E-07	4.00E-07	4.20E-07	4.00E-08	6.50E-07	0.128 ± 0.019	0.228 ± 0.012	0.025 ± 0.010	0.241 ± 0.025
**TPD**	5.93E-01	5.51E + 00	9.75E + 01	5.66E-01	5.92E + 01	0.004 ± 0.001	0.034 ± 0.007	0.003 ± 0.006	0.597 ± 0.023
**NVD**	3.37E-01	1.53E + 00	6.21E + 00	5.60E-01	9.53E + 00	0.019 ± 0.004	0.084 ± 0.009	0.031 ± 0.011	0.342 ± 0.027
**TPV**	8.18E-02	4.93E-01	1.90E + 00	1.37E-01	2.66E + 00	0.016 ± 0.004	0.094 ± 0.009	0.026 ± 0.010	0.361 ± 0.026
**FPV**	1.40E-04	7.90E-04	2.22E-03	2.20E-04	3.73E-03	0.020 ± 0.004	0.111 ± 0.010	0.031 ± 0.011	0.312 ± 0.026
**FR**	5.00E-07	2.60E-06	4.21E-05	4.00E-07	2.22E-05	0.007 ± 0.002	0.039 ± 0.007	0.005 ± 0.007	0.621 ± 0.023

σ^2^  _a_, additive genetic variance; σ^2^_m_, maternal genetic variance_;_ σ^2^_p_, pen variance; σ^2^_c_, common litter genetic variance; σ^2^_e_, residual variance; $c_{p}^{2}$, proportion of variance explained by pen effects; $c_{m}^{2}$, proportion of variance explained by maternal effects; $c_{c}^{2}$, proportion of variance explained by common litter effects and

ADG120, average daily gain; BF120, backfat thickness; LD120, loin depth; DFI, daily feed intake; FCR, feed conversion ratio; FE, feed efficiency; FI, feed intake; FPV, feed intake per visit; FR, feed intake rate; KR, Kleiber ratio; NVD, number of visits to feeder per day; RFI, residual feed intake (1-6); RG, residual gain; RIG, residual intake and gain; TPD, total time spent eating per day; TPV, time spent eating per visit, FR, feeding rate.

Previous studies (Rauw et al. 2006; Lu et al. 2017) indicated that a random pen effect may be important when estimating genetic parameters for feed efficiency and feeding behavior. The significant random effect of pen was reported in several studies for feed efficiency in pigs (Do et al. 2013; Lu et al. 2017; Santiago et al. 2021b). The phenotypic variances of feed efficiency traits varied among breeds and the methods of using pen as a random effect (physical space versus social interaction group). For instance, Lu et al. (2017) reported that the variances in Gain to Feed ratio (G: F) and six different measures of RFI explained by pen (social interaction group) ranged from 14% to 17%. In contrast, Do et al. (2013) reported a lower percentage variance attributed to common pen effects, with estimates ranging from 3% to 5% for FCR and RFI1, and from 2% to 4% for RFI2 across three different pig breeds. In the current study, pen also explained a small proportion of the phenotypic variance for ADG120 and BF120, aligning with findings by Do et al. (2013), who reported that pen accounted for 3% to 5% of the phenotypic variance in ADG120 and 3–6% in BF across three Danish pig breeds. Regarding feeding behaviors, Santiago et al. (2021a) reported that the common spatial pen effect explained between 0.3% and 23% of the total phenotypic variance of feeding behavior traits. Similarly, Do et al. (2013) reported a substantial percentage of variance explained by pen in different feeding behavior traits for Duroc pigs, including 4% for DFI, 3% for TPD, 12% for TPV, 5% for FR, 9% for NVD, and 10% for FPV.

Common litter effect was reported as a significant factor influencing feed efficiency traits (Johnson et al. 1999; Hoque et al. 2009). However, in the current study, these effects only explained less than 5% of the phenotypic variance in most feed efficiency traits, consistent with the findings of Lu et al. (2017). Due to their relatively minor impact, common litter effects were excluded from several earlier studies modeling RFI (Do et al. 2013; Santiago et al. 2021a). Santiago et al. (2021b) reported that the extent to which common litter effects accounted for variance in feeding behavior traits varied across different models. For example, in a model incorporating only additive and common litter effects, the common litter effect explained 14% of the variance in DFI, 7% in TPV, 4% in NVD, and 11% in FPV, but had no explanatory power for TPD and FR. Maternal genetic effects also explained a small proportion of phenotypic variance of feed efficiency measures, though their influence was more pronounced for ADG120. It remains unclear how the maternal genetic effects contributed a significant amount of phenotypic variance of ADG120 and KR. To the best of our knowledge, none of the previous RFI studies in pigs has reported the effects of maternal genetics on these traits.

Moderate to high heritability estimates for the production traits in the current study are consistent with many previous reports. For instance, the moderate heritability of ADG120 in Duroc pigs was also reported by Do et al. (2013) and Lu et al. (2017). Similarly, high heritability for BF120 was also reported in Danish pigs (Do et al. 2013), American pigs (Lu et al. 2017), Canadian pigs (Miar et al. 2014a,b) and Korean pigs (Santiago et al. 2021b). Moderate heritability estimates for RFIs, FCR, RIG, and RG in this study were largely in agreement with findings from other studies. Specifically, heritability estimates for RFI (0.27–0.29) fall within the previously reported range of 0.10 to 0.51 reported in previous research (Hoque et al. 2009; Jiao et al. 2014). FCR was similarly reported to be moderately heritable by other studies (Hoque et al. 2009; Do et al. 2013; Lu et al. 2017; Homma et al. 2021; Santiago et al. 2021b). In addition, Lu et al. (2017) reported a higher heritability estimate for RIG (0.57) in Duroc pigs raised in the USA compared to that reported in the current study. Moderate heritability estimated for DFI was also reported in many other studies of Duroc pigs, with estimates of 0.41 by Do et al. (2013) and 0.44 by Hoque and Suzuki (2008). For other feeding behavior traits, moderate to high heritability was also observed in other studies (Do et al. 2013; Lu et al. 2017; Santiago et al. 2021a). In particular, TPD was a highly heritable trait in the current study, which was in agreement with the estimates observed by Do et al. (2013) (0.56) or Lu et al. (2017) (ranging from 0.51 to 0.71). Heritability estimates for FPV (0.66) and NVD (0.53) were similar to those reported by Langdon (2015) and Do et al. (2013). Notably, while most previous studies included only boars in their models, the current study incorporated data from both sexes. Since heritability can vary by breed, sex, and environmental context, eg, higher estimates in Duroc vs. Landrace pigs (Santiago et al. 2021b) highlighting the need for breed-specific models to avoid biased predictions in multi-breed populations. These heritability estimates underscore the genetic basis of feed efficiency and feeding behaviors in pigs, enabling targeted breeding programs to improve traits. The moderate to high heritability for behavioral traits, such as TPD and FPV (0.51–0.71) suggests strong potential for genomic selection, as these values indicate that 51–71% of phenotypic variation is attributable to additive genetic effects, with the remainder influenced by environmental factors like housing density or feed access. Selecting for efficient behaviors might get a quick response due to moderate to high heritability of the traits and could be used to indirectly enhance overall feed conversion while reducing energy expenditure on non-productive activities like excessive aggression at feeders.

### Phenotypic and genetic correlations among the studied traits

#### Feed efficiency and growth performance

Genetic and phenotypic correlations of the traits are shown in Table 4. Genetic correlations between different RFI measures and ADG120 varied significantly. RFI1 and RFI3 had significant negative genetic correlations with ADG120 (-0.56 ± 0.08 and -0.45 ± 0.09, respectively), whereas RFI2, RFI4, and RFI5 had near-zero and nonsignificant genetic correlations. RFI6 showed a low but significant genetic correlation with ADG120 (0.15 ± 0.07). All RFI measures had positive genetic correlations with BF120, ranging from 0.13 ± 0.06 for RFI6 to 0.58 ± 0.04 for RFI1 and RFI2. Significant negative genetic correlations with LD120 were found for RFI1, RFI2, and RFI3 (-0.40 ± 0.05, -0.41 ± 0.05, and -0.23 ± 0.06, respectively), while RFI4, RFI5, and RFI6 exhibited weak and nonsignificant correlations.

**Table 4:** Estimates of genetic correlations (*above diagonal*), and phenotypic correlations (*below diagonal*) between feed efficiency, growth and feeding behavior traits.

Trait*	ADG120	BF120	LD120	FCR	RFI1	RFI2	RFI3	RFI4	RFI5	RFI6	RG	RIG	KR	DFI	TPD	NVD	TPV	FR	FPV
**ADG120**		**0.15±0.01**	**-0.26±0.02**	**-0.64±0.07**	**-0.56 ± 0.08**	0.01 ± 0.11	**-0.45 ± 0.09**	0.01 ± 0.11	-0.04 ± 0.08	**0.15 ± 0.07**	**0.68 ± 0.04**	**0.23 ± 0.06**	**0.87 ± 0.03**	**0.13 ± 0.02**	**-0.42 ± 0.14**	**0.12 ± 0.07**	**-0.7 ± 0.07**	**-0.11 ± 0.05**	0.02 ± 0.07
**BF120**	**0.18 ± 0.06**		**-0.46 ± 0.06**	**0.55 ± 0.05**	**0.58 ± 0.04**	**0.58 ± 0.04**	**0.28 ± 0.05**	**0.17 ± 0.06**	**0.27 ± 0.05**	**0.13 ± 0.06**	0.00 ± 0.04	**-0.23 ± 0.06**	-0.01 ± 0.07	**0.38 ± 0.01**	0.04 ± 0.04	-0.04 ± 0.06	0.02 ± 0.06	**-0.25 ± 0.05**	**0.13 ± 0.06**
**LD120**	0.07 ± 0.08	**-0.26 ± 0.01**		**-0.61 ± 0.07**	**-0.40 ± 0.05**	**-0.41 ± 0.05**	**-0.23 ± 0.06**	0.01 ± 0.05	**-0.12 ± 0.08**	-0.04 ± 0.08	-0.07 ± 0.30	0.09 ± 0.08	-0.02 ± 0.11	**-0.30 ± 0.02**	-0.10 ± 0.06	-0.12 ± 0.08	-0.01 ± 0.07	0.03 ± 0.02	**0.05 ± 0.01**
**FCR**	**-0.49±0.02**	**0.39 ± 0.01**	**-0.21 ± 0.02**		**0.97 ± 0.01**	**0.97 ± 0.01**	**0.89 ± 0.03**	**0.78 ± 0.05**	**0.88 ± 0.03**	**0.32 ± 0.09**	**-0.15 ± 0.03**	**-0.89 ± 0.01**	-0.06 ± 0.11	**0.35±0.06**	**0.05 ± 0.01**	**0.09 ± 0.01**	**-0.10 ± 0.02**	0.09 ± 0.05	0.09 ± 0.18
**RFI1**	**-0.11 ± 0.01**	**0.42 ± 0.01**	**-0.18 ± 0.02**	**0.49 ± 0.17**		**0.79 ± 0.03**	**0.52 ± 0.1**	**0.83 ± 0.04**	**0.60 ± 0.07**	**0.97 ± 0.01**	**-0.98 ± 0.02**	**-0.89 ± 0.02**	-0.05 ± 0.10	**0.93±0.01**	-0.09 ± 0.05	**0.15 ± 0.06**	-0.11 ± 0.09	**0.25 ± 0.03**	0.07 ± 0.05
**RFI2**	**0.06 ± 0.01**	**0.42 ± 0.01**	**-0.20 ± 0.02**	**0.36 ± 0.12**	**0.63 ± 0.09**		**0.8 ± 0.02**	**0.64 ± 0.05**	**0.77 ± 0.03**	**0.70 ± 0.04**	**-0.93 ± 0.02**	**-0.89 ± 0.02**	-0.03 ± 0.06	**0.90 ± 0.13**	-0.08 ± 0.05	0.14 ± 0.06	-0.14 ± 0.08	**0.22 ± 0.06**	0.08 ± 0.06
**RFI3**	**-0.06 ± 0.01**	**0.14 ± 0.01**	**-0.10 ± 0.02**	**0.34 ± 0.13**	**0.52 ± 0.06**	**0.67 ± 0.09**		**0.83 ± 0.02**	**0.79 ± 0.06**	**0.78 ± 0.03**	**-0.91 ± 0.02**	**-0.96 ± 0.01**	-0.06 ± 0.11	**0.91 ± 0.11**	**-0.11 ± 0.05**	**0.17 ± 0.06**	**-0.15 ± 0.06**	**0.18 ± 0.09**	**0.31 ± 0.05**
**RFI4**	**0.05 ± 0.01**	**0.05 ± 0.01**	-0.02 ± 0.02	**0.31 ± 0.09**	**0.79 ± 0.16**	**0.82 ± 0.02**	**0.82 ± 0.13**		**0.48 ± 0.20**	**0.68 ± 0.17**	**-0.92 ± 0.01**	**-0.94 ± 0.01**	-0.03 ± 0.06	**0.84 ± 0.11**	**-0.11 ± 0.05**	**-0.11 ± 0.05**	**0.17 ± 0.06**	**-0.15 ± 0.06**	**0.18 ± 0.09**
**RFI5**	**-0.13 ± 0.01**	**0.12 ± 0.01**	**0.12 ± 0.01**	**0.34 ± 0.13**	**0.71 ± 0.24**	**0.71 ± 0.19**	**0.74 ± 0.06**	**0.79 ± 0.26**		**0.72 ± 0.07**	**-0.91 ± 0.02**	**-0.96 ± 0.01**	-0.09 ± 0.09	**0.88 ± 0.01**	**-0.12 ± 0.05**	**0.17 ± 0.07**	0.01 ± 0.04	**0.20 ± 0.03**	-0.47 ± 0.26
**RFI6**	**0.07 ± 0.01**	**0.03 ± 0.01**	**0.05 ± 0.01**	**0.32 ± 0.09**	**0.73 ± 0.15**	**0.81 ± 0.18**	**0.81 ± 0.03**	**0.51 ± 0.11**	**0.71 ± 0.11**		**-0.74 ± 0.04**	**-0.94 ± 0.01**	-0.09 ± 0.09	**0.84 ± 0.01**	**-0.12 ± 0.05**	**0.16 ± 0.07**	**-0.16 ± 0.06**	**0.30 ± 0.02**	0.03 ± 0.06
**RG**	**0.83 ± 0.1**	**-0.06 ± 0.02**	-0.05 ± 0.05	**-0.15 ± 0.03**	**-0.32 ± 0.03**	**-0.58 ± 0.03**	**-0.29 ± 0.03**	**-0.76 ± 0.01**	**-0.47 ± 0.03**	**-0.49 ± 0.03**		**0.22 ± 0.09**	-0.46 ± 1.12	**-0.35 ± 0.01**	**-0.16 ± 0.08**	**-0.31 ± 0.09**	0.05 ± 0.06	-0.05 ± 0.09	**-0.48 ± 0.03**
**RIG**	**0.42 ± 0.01**	**-0.10 ± 0.01**	**-0.03 ± 0.01**	**-0.89 ± 0.21**	**-0.80 ± 0.01**	**-0.80 ± 0.01**	**-0.84 ± 0.01**	**-0.81 ± 0.01**	**-0.74 ± 0.02**	**-0.75 ± 0.01**	**0.76 ± 0.01**		-0.05 ± 0.14	**-0.76 ± 0.01**	**0.17 ± 0.05**	**-0.14 ± 0.06**	**0.17 ± 0.06**	**-0.38 ± 0.04**	-0.09 ± 0.06
**KR**	**0.72 ± 0.03**	-0.04 ± 0.05	-0.02 ± 0.04	0.05 ± 0.11	**-0.24 ± 0.25**	-0.23 ± 0.24	**-0.25 ± 0.26**	**0.02 ± 0.01**	-0.29 ± 0.42	-0.29 ± 0.42	**0.89 ± 0.01**	0.03 ± 0.02		**-0.03 ± 0.01**	**0.93 ± 0.17**	**0.82 ± 0.23**	-0.11 ± 0.29	-0.47 ± 0.59	**-0.14 ± 0.05**
**DFI**	**0.15 ± 0.06**	**0.58 ± 0.05**	**-0.50 ± 0.08**	**0.84±0.04**	**0.94±0.01**	**0.79 ± 0.03**	**0.52 ± 0.10**	**0.83 ± 0.04**	**0.60 ± 0.07**	**0.97 ± 0.01**	**-0.44 ± 0.05**	**-0.89 ± 0.02**	-0.05 ± 0.10		-0.09 ± 0.05	**0.15 ± 0.06**	-0.11 ± 0.09	**0.20 ± 0.06**	**0.12 ± 0.06**
**TPD**	**-0.19 ± 0.07**	**0.03 ± 0.01**	**-0.07 ± 0.01**	**0.03 ± 0.01**	0.01 ± 0.01	**0.03 ± 0.01**	0.01 ± 0.01	**0.11 ± 0.01**	**0.15 ± 0.01**	**0.02 ± 0.01**	**0.02 ± 0.01**	**0.06 ± 0.01**	-0.05 ± 0.14	**0.03 ± 0.01**		**0.21 ± 0.05**	**0.52 ± 0.04**	**0.12 ± 0.04**	0.01 ± 0.04
**NVD**	**0.04 ± 0.01**	**-0.04 ± 0.01**	-0.01 ± 0.01	**0.09 ± 0.01**	**0.09 ± 0.01**	**0.08 ± 0.01**	**0.11 ± 0.01**	**-0.05 ± 0.01**	**0.10 ± 0.01**	**0.12 ± 0.01**	**0.03 ± 0.01**	**-0.06 ± 0.01**	**0.72 ± 0.05**	**0.05 ± 0.01**	**0.21 ± 0.05**		**-0.73 ± 0.03**	**0.33 ± 0.04**	0.03 ± 0.23
**TPV**	**-0.21 ± 0.02**	**0.03 ± 0.01**	**-0.03 ± 0.01**	**-0.10 ± 0.02**	**-0.08 ± 0.02**	**-0.08 ± 0.02**	**-0.04 ± 0.02**	**0.36 ± 0.12**	**-0.05 ± 0.02**	**-0.05 ± 0.01**	**0.03 ± 0.01**	**0.06 ± 0.01**	0.00 ± 0.11	-0.01 ± 0.01	**0.52 ± 0.04**	**-0.73 ± 0.03**		**0.13 ± 0.04**	**0.14 ± 0.03**
**FR**	**-0.08 ± 0.01**	**-0.24 ± 0.04**	**0.08 ± 0.01**	**0.09 ± 0.05**	0.03 ± 0.05	0.04 ± 0.05	0.04 ± 0.05	**-0.52 ± 0.03**	-0.01 ± 0.06	**0.09 ± 0.04**	**-0.28 ± 0.01**	**-0.33 ± 0.01**	0.03 ± 0.06	**0.11 ± 0.04**	**0.11 ± 0.04**	**0.33 ± 0.04**	**0.15 ± 0.04**		**0.24 ± 0.03**
**FPV**	**0.13 ± 0.01**	**0.11 ± 0.01**	**0.06 ± 0.01**	0.09 ± 0.18	**0.18 ± 0.01**	0.09 ± 0.17	0.09 ± 0.18	0.09 ± 0.11	0.09 ± 0.18	**-0.20 ± 0.02**	**-0.14 ± 0.01**	**-0.15 ± 0.01**	**-0.45 ± 0.04**	**0.20 ± 0.01**	0.01 ± 0.04	0.03 ± 0.23	**0.13 ± 0.03**	**0.21 ± 0.03**	

* ADG120, average daily gain; BF120, backfat thickness; LD120, loin depth; DFI, daily feed intake; FCR, feed conversion ratio; FE, feed efficiency; FI, feed intake; FPV, feed intake per visit; FR, feed intake rate; KR, Kleiber ratio; NVD, number of visits to feeder per day; RFI, residual feed intake (1-6); RG, residual gain; RIG, residual intake and gain; TPD, total time spent eating per day; TPV, time spent eating per visit.

Bold indicated the correlations were significantly differed from zero.

FCR had significant phenotypic and genetic correlations with ADG120 (-0.49 ± 0.02 and -0.64 ± 0.07, respectively), BF120 (0.55 ± 0.01 and 0.39 ± 0.01, respectively), and LD (-0.21 ± 0.02 and -0.61 ± 0.07, respectively). Both RG and RIG had significantly positive phenotypic and genetic correlations with ADG120 (0.83 ± 0.10 and 0.68 ± 0.01 and 0.23 ± 0.06 and 0.42 ± 0.01, respectively). High genetic correlations were also observed among RFI measures, ranging from 0.52 ± 0.10 for RFI1 and RFI3, to 0.97 ± 0.01 for RFI1 and RFI6. FCR showed significant moderate to high genetic correlations with all RFI measures from 0.32 ± 0.09 (with RFI6) to 0.97 ± 0.01 (with RFI2). Significant negative genetic correlations were observed for all RFI measures with RG and RIG.

KR had no significant genetic correlation with BF120 and LD120. As anticipated, the estimated phenotypic correlations of RFIs with other covariates in the models were close to zero. Our results aligned with the findings of Kennedy et al. (1993), who suggested RFI is phenotypically independent of production traits but might be genetically correlated to production. In this study, genetic correlations between RFIs and other covariates in the RFI models were not always close to zero, consistent with findings in different breeds (de Haer et al. 1993; Johnson et al. 1999; Baumung et al. 2006; Hoque et al. 2009). For instance, Johnson et al. (1999) reported similar estimates of genetic correlations of RFIs with ADG (0.17) and a slightly positive genetic correlation of RFI2 with BF (0.22) in Large White boars under individual pen testing. Nguyen et al. (2005) also reported a slightly negative genetic correlation between RFI (adjusted for test ADG and BF) and BF (-0.20). Apart from sampling errors, differences in these estimates can result from population differences in phenotypic and genetic correlations among traits.

High and positive genetic correlations between RG and ADG120 (0.68 ± 0.04) reported in the current study agreed with the results by Berry and Crowley (2013), who reported a positive high genetic correlation between RG and ADG (0.82) in cattle. Similarly, the strong and significant genetic correlation between KR and ADG120 (0.87 ± 0.03) in this study aligns with findings from other studies. For instance, Steyn et al. (2014) and Crowley et al. (2010) reported high genetic correlations of 0.91 and 0.75 in cattle, respectively, while Mandal et al. (2015) found a correlation of 0.91 in sheep. Since both traits are linked to how efficiently an animal converts feed into body mass, pigs with higher metabolic rates also have higher ADG120 and higher KR. Berry and Crowley (2013) also observed a nonsignificant pooled genetic correlation of -0.04 between KR and DFI, which aligns with our results. In other species, KR has been proposed as an alternative indicator for feed efficiency (e.g. Davoudi et al. 2022), particularly where measuring feed efficiency is challenging to automate and apply on a large scale. However, this may not be applicable to the pig industry.

#### Feed efficiency and feeding behavior traits

In the current study, we reported strong genetic correlations between DFI and all feed efficiency traits (Table 4). DFI had positive genetic correlations with FCR (0.35 ± 0.06) and RFI measures (from 0.84 ± 0.11 to 0.93 ± 0.01), while it had negative genetic correlations with RG (-0.35 ± 0.01) and RIG (-0.76 ± 0.01). Some feed efficiency traits had weak and non-significant genetic correlations with other feeding behavior traits (Table 4). Genetic correlations between RFIs and TPD ranged from -0.11 (with RFI3 and RFI4) to -0.08 (with RFI2). FCR also showed very weak to negligible genetic correlations with feeding behavior traits, such as TPD (0.05 ± 0.01), NVD (0.09 ± 0.01), TPV (-0.10 ± 0.02), FR (0.09 ± 0.05), and FPV (0.09 ± 0.18). However, significant and moderate genetic correlations were observed between RG and NVD (-0.31 ± 0.09) and between RIG and FR (-0.38 ± 0.04).

DFI was strongly correlated with all the measurements of RFIs, both genetically and phenotypically, suggesting that selection against RFI may cause a reduction in DFI (Hoque et al. 2009). FCR exhibited a favorable genetic correlation to DFI, though the magnitude of the correlation we found was lower compared to RFI, which is in agreement with previous studies (Do et al. 2013; Santiago et al. 2021a). Similarly, Cai et al. (2008) also suggested that selection for low RFI in pigs led to a significant reduction in DFI.

The weak to moderate genetic correlations observed between feed efficiency traits and feeding behavior traits in this study are consistent with previous research (VonFelde et al. 1996; Labroue et al. 1997; Schulze et al. 2003; Rauw et al. 2006; Young et al. 2011; Lu et al. 2017; Santiago et al. 2021a). For instance, Santiago et al. (2021a) reported low to moderate positive genetic correlations of TPD and NVD with feed efficiency traits in Duroc pigs, including FCR (0.27 and 0.34), RFI1 (0.33 and 0.46), RFI2 (0.24 and 0.38), RFI3 (0.33 and 0.47), RFI4 (0.24 and 0.39), and RFI5 (0.24 and 0.39), respectively. Based on these findings, the authors proposed that selection against TPD would likely improve FCR and RFI with minimal impact on ADG and only a slight reduction in BF.

In the current study, TPV had very low or negligible genetic correlation with RFIs, aligning with earlier estimates reported by Von Felde et al. (1996). Similarly, FCR had weak and positive genetic correlations with NVD and TPD, and a weak negative genetic correlation with TPV (-0.10 ± 0.02), which were also reported in other studies (Labroue et al. 1997; Schulze et al. 2003).

#### Growth performance and feeding behaviors

Positive and significant genetic correlations were found between ADG120 and BF120 (0.15 ± 0.01), ADG120 and DFI (0.13 ± 0.02), and BF120 and DFI (0.38 ± 0.01) in the current study (Table 4). Previous research has found that in general, faster-growing animals tend to consume more feed and accumulate more fat (de Haer and de Vries 1993; Mrode and Kennedy 1993; Rauw et al. 2006). Genetic correlations between ADG120 and feeding behaviors varied, as ADG120 had a strong negative genetic correlation with TPV (-0.70 ± 0.07) but moderate negative genetic correlation with TPD (-0.42 ± 0.14) in the current study (Table 4). BF120 and LD120 had very low genetic correlations with other feeding behavior traits, ranging from 0.04 ± 0.06 (BF120 and NVD) to -0.25 ± 0.0 (BF120 and FR), and from -0.01 ± 0.07 (LD120 and TPV) to -0.12 ± 0.08 (LD120 and NVD), as highlighted in Table 4. These findings are in agreement with Von Felde et al. (1996), who reported no genetic correlation between NVD and performance traits. Similarly, Lu et al. (2017) reported a near-zero genetic correlation between TPD and on-test ADG (0.11 ± 0.04), while Do et al. (2013) reported a low positive genetic correlation (0.23 ± 0.09). Additionally, Lu et al. (2017) reported a low negative genetic correlation between NVD and BF (-0.25 ± 0.08). Moderate genetic correlations between NVD and on-test ADG (-0.26 ± 0.09 and -0.28 ± 0.03) were reported by both Do et al. (2013) and Lu et al. (2017).

In the current study, most feeding behavior traits exhibited weak genetic correlations with one another, with a few notable exceptions. Moderate genetic correlations were observed between TPV and TPD (0.52 ± 0.04), TPV and NVD (-0.73 ± 0.03), NVD and FPV (-0.89 ± 0.11), and TPV and FR (0.33 ± 0.04) (Table 4). High negative genetic and phenotypic correlations between NVD and both TPD and FPV were in agreement with findings from previous studies in Duroc pigs (Do et al. 2013; Santiago et al. 2021a). In addition, Labroue et al. (1997) found high genetic correlations between TPV and FPV (0.83 ± 0.04 and 0.87 ± 0.02), FPV and NVD (-0.93 ± 0.02 and -0.94 ± 0.02), and TPD and FIR (-0.86 ± 0.03 and -0.78 ± 0.04) in French Landrace and Large White breeds, respectively. Moderate to high genetic correlations between ADG and BF, ADG and DFI, and BF and DFI in this study and other previous studies suggest animals with faster growth rates tend to deposit more fat and have greater feed intake (de Haer and de Vries 1993; Mrode and Kennedy 1993; Rauw et al. 2006). In contrast, genetic correlations between TPD or NVD and growth and performance traits, such as BF and ADG were typically low or near-zero in previous studies (VonFelde et al. 1996; Do et al. 2013; Lu et al. 2017). For instance, Lu et al. (2017) reported low genetic correlations between NVD and BF (-0.25 ± 0.08) and between TPD and BF (0.17 ± 0.02), while VonFelde et al. (1996) found that NVD had no genetic correlation with performance traits.

Selection for improved feed efficiency using either FCR or RFI depends on the breeding objectives. Since selection for FCR is helping convert feed into body mass, it might be suitable for systems where the primary objective is rapid growth (high ADG). In contrast, RFI is better suited for systems where the breeding objective aims to enhance overall feed efficiency without compromising growth or other performance traits.

Notably, genetic correlations between FCR and RFI are high and positive, ranging from 0.53 to 0.95 (Hoque et al. 2009; Jiao et al. 2014), indicating that selection for one is likely to impact the other. Several studies reported that FCR had moderate positive correlations with body weight and ADG (Hoque et al. 2009; Hong et al. 2021), indicating that FCR can be indirectly altered through the selection of growth traits. Conversely, selecting for growth traits, such as body weight, ADG, and sometimes BF does not affect or has a minor impact on RFI, as it is calculated based on the regression of feed intake on growth traits (Koch et al. 1963; Hoque et al. 2007; Saintilan et al. 2011).

The genetic correlations between RFI and carcass traits, such as BF are typically low and close to zero (Hoque et al. 2007; Saintilan et al. 2011). In addition, RFI tends to be moderately and negatively correlated with body leanness, as reported in previous studies (Cai et al. 2008; Hsu et al. 2015), suggesting that selecting for low RFI may enhance lean growth. Moreover, pigs with low RFI are preferred as they use less energy for feed intake, social interactions, heat production, and maintenance (Gilbert et al. 2017). Finally, RFI is highly correlated with DFI, meaning that selecting for lower RFI can reduce DFI (Gilbert et al. 2017). Although FCR is positively correlated with DFI, this correlation is weaker than that of RFI, thus, selecting for low RFI would reduce DFI more effectively than selecting based on FCR in pig breeding programs.

Feeding behavior traits have been suggested as auxiliary indicators for improving feed efficiency and production traits in pigs (Santiago et al. 2021a). Inclusion of the feeding behavior traits into selection indices was shown to increase the selection response for feed efficiency traits, such as FCR (4%) and RFI (1.8%) in Pietrain pigs (Núñez et al. 2023). Additionally, changes in feeding behaviors are considered as one of the first clinical signs when animals are exposed to stressors, such as disease (Cheng et al. 2021). In fact, Putz et al. (2019) showed that day-to-day variations in feed intake and feeding behavior (duration) can be used as new phenotypes for assessing resilience in weaning-to-finishing pigs.

Heritability estimates for feeding behavior traits are moderate to high, suggesting their potential responsiveness to selective breeding programs. However, their diverse genetic correlations with feed efficiency, production, and carcass traits indicate that including them in breeding programs requires careful consideration and setup. Clear breeding objectives, understanding the genetic architecture of these traits, and evaluating the potential trade-offs are required to ensure the overall improvement of pig performance and welfare.

## Conclusion

The results of the current study indicate improving feed efficiency through selection for lower RFI can help lower feed costs of swine production with little impact on growth and carcass traits in Canadian Duroc pigs. Feeding behavior traits show moderate to high heritability; however, their weak and variable genetic correlations with feed efficiency, growth, and carcass traits in the current study suggest that their inclusion in selection programs requires careful evaluation of economic relevance and potential trade-offs. Future research should aim to use genomic tools to increase the accuracy of predicting RFI and related traits and to study the biological processes, such as gene expression and metabolism, that drive feeding behavior and efficiency.

## Abbreviations

A120: ages adjusted to 120 kg of body weight
ADG120: average daily gain adjusted to 120 kg of body weight
BW: body weight
BF120: backfat thickness adjusted to 120kg of body weight
DFI: daily feed intake
FCR: feed conversion ratio
FE: feed efficiency
FI: feed intake
FPV: feed intake per visit
FR: feed intake rate
KR: Kleiber ratio
LD120: loin depth adjusted for 120 kg of body weight
NVD: number of visits to the feeder per day
RFI: residual feed intake
RG: residual gain
RIG: residual intake and gain
TPD: total time spent eating per day
TPV: time spent eating per visit
